# Reanalysis of the Bridge et al. study of suicide following release of *13 Reasons Why*

**DOI:** 10.1371/journal.pone.0227545

**Published:** 2020-01-16

**Authors:** Daniel Romer

**Affiliations:** Annenberg Public Policy Center, University of Pennsylvania, Philadelphia, Pennsylvania, United States of America; Medical University of Vienna, AUSTRIA

## Abstract

Bridge et al. recently presented a time series analysis of suicide rates in the US following the release of the 2017 Netflix series “13 Reasons Why.” Their analysis found a powerful effect of the show on boys ages 10–17 for nine months after the show was released in April 2017. I questioned this finding on two grounds. First, contagion would be expected to be stronger for girls than boys for this story, and second their analysis did not take into account strong secular trends in suicide, especially in boys from 2016 to 2017. I reanalyzed their data using a simple auto-regression model that tested for changes in rates after removing auto-correlation and national trends in suicide. I found that the increase for boys observed by Bridge et al. in April was no greater than the increase observed during the prior month before the show was released. There were also no effects in later months of that year. For girls, I found a small but nonsignificant increase in suicide in April that was unique to that month, potentially consistent with a combined protective and harmful effect of the show. In total, I conclude that it is difficult to attribute harmful effects of the show using aggregate rates of monthly suicide rates. More fine-grained analyses at the weekly level may be more valid but only after controlling for secular changes in suicide that have been particularly strong since 2008 in the US.

## Introduction

A recent analysis [1] found an increase in aggregate suicide rates among youth ages 10 to 17 in the months following the release of the first season of the Netflix series “13 Reasons Why” (13RW). This increase was surprising in that it was unique to male youth when the show focused on the suicide death of a high school girl and the many classmates whom she blamed for instigating her death. I reexamine the finding with regard to what can constitute evidence of contagion and how the recent trends in suicide in youth need to be considered when attributing changes in suicide rates to media events such as 13RW. A reanalysis controlling for those trends finds no evidence for boys and the possibility of an effect for girls.

### Contagion effects on suicide

The evidence for suicide contagion is strong especially for news reporting [2], but many instances of contagion from fictional portrayals have also been observed. Indeed, the classic case of such contagion led to the coining of the label, the Werther effect. In “The Sorrows of Young Werther”, a novel written in 1774 by the up-and-coming German writer Johann Wolfgang von Goethe, a young man ends his life on Christmas Eve after being rejected by his love interest and experiencing other humiliations. The story was said to trigger suicides among young men of a similar age at the time and led to the banning of the book in some countries [3].

A more recent example of the Werther effect in a fictional account is the 1980’s German television series “Tod eines Schülers” (Death of a Student), in which a young man again was the focus of the story [4]. In this and other examples, the basis for the conclusion that contagion was present is (a) the coincidence in time of the increase in suicide events (b) among persons with similar demographic characteristics, and (c) if possible, evidence of the use of similar methods. For example, [5] found that a television show that portrayed a self-poisoning by a young male protagonist led to an increase in hospital admissions two weeks after the show using a similar method by both men and women of a similar age. This type of evidence makes it difficult to attribute the increase to other sources of contagion or to other events that might lead to changes in suicide attempts.

In contrast to earlier studies of contagion, the Bridge et al. study presented evidence only of an increase in boys in the months after the show’s April 2017 release. Surprisingly, the effect persisted throughout the year, suggesting a very powerful contagion effect among boys but not girls. Another study found an increase in hospital admissions for various forms of self-injury suggestive of contagion following the show’s release but no evidence of the use of similar methods or similar gender [6].

### Possible benefits of suicide portrayals

Although Bridge et al. could not identify contagion in girls, there is reason to believe that such an effect might be muted by the presence of another phenomenon known as the Papageno effect [7], after a character in Mozart’s “The Magic Flute.” In this form of media contagion, a story might convey greater understanding of those in a suicidal crisis and provide vulnerable audience members with some perspective about how a person’s suicide might impact one’s community. This form of empathy, known as perspective taking, can be an outcome of a fictional story [8]. And it may produce a decrease in suicide among those exposed to it [9].

In a recent study, we found that for the second season of 13RW, some of the more vulnerable viewers who were able to see the entire second season came away with a beneficial response [9]. These viewers were less immediately suicidal after the show than those who did not watch at all, and they were more sympathetic to helping a person in a suicidal crisis than those who either stopped watching or did not watch at all. These findings suggested the potential for Papageno effects for some viewers of 13RW.

At the same time, there were also harmful effects of the show for those who stopped watching, especially if they were vulnerable to suicide to begin with. We interpreted that finding as consistent with a Werther effect because the sample was primarily young women, as was the show’s victim of suicide. It is possible therefore that for the first season there were young women who benefited from the show, which had the effect of muting the overall Werther effect and making it difficult to detect.

**Fig 1:**
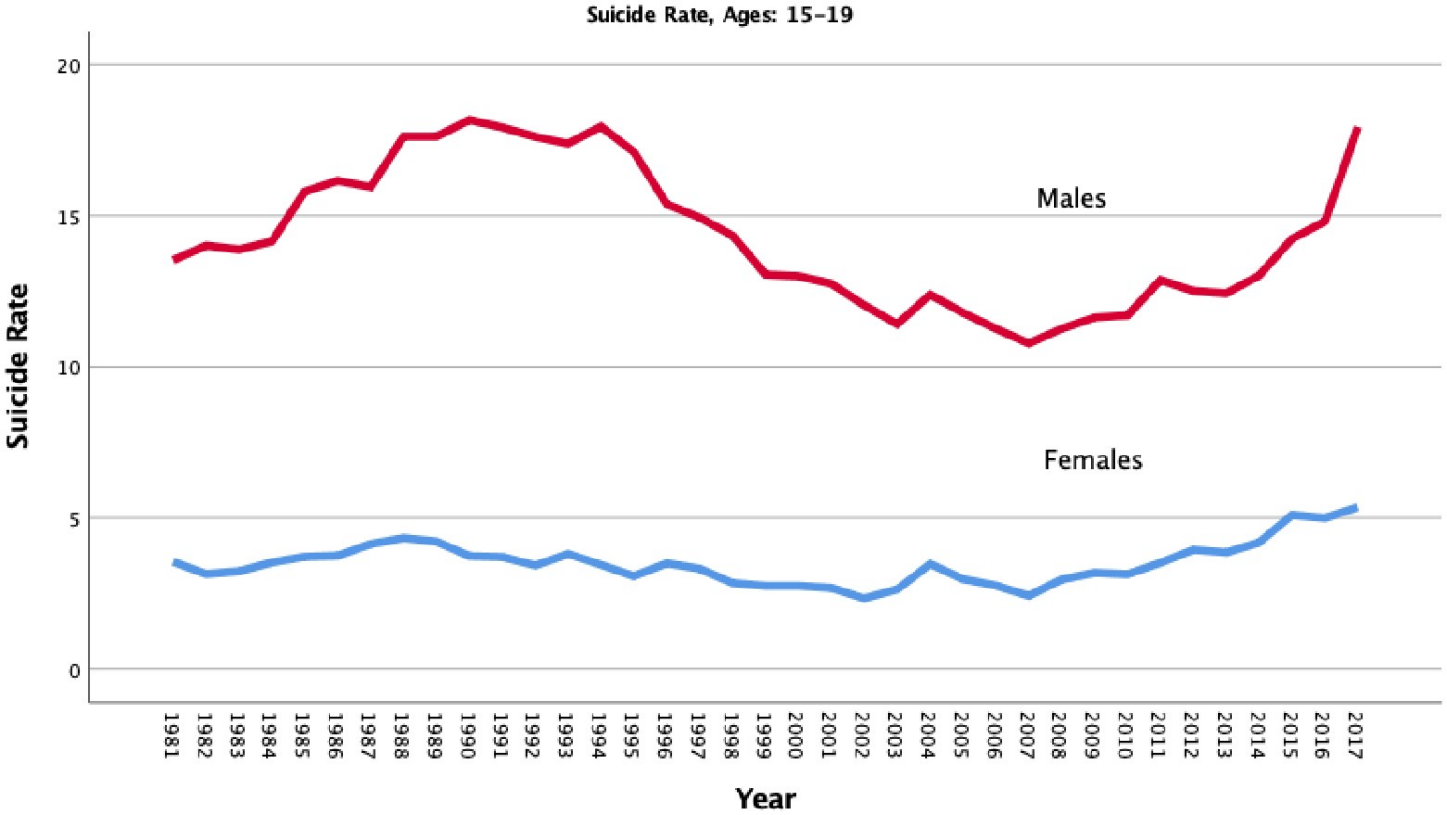
Suicide rates per 100,000 in youth ages 15–19 in the US, 1981–2017. Source: CDC.

In the case of young men, there would not be reason to expect much of a Werther effect, especially in the first season where the young female protagonist was the major source of potential contagion. Which is why I was led to reanalyze the Bridge et al. findings. I was also concerned because suicide rates in young people ages 15–19 have been rising since the financial crisis of 2008 and rose by 20% from 2016 to 2017 in young men (Fig 1). This was a particularly large increase that was unlikely to be the result of a single television show. Unless an analysis controlled for this trend, it would be difficult to separate the effects of the show from the secular trend.

## Method

The study [1] examined US national monthly suicide rates from January 1, 2013 to December 31, 2017 for three age groups: 10–17, 18–29, and 30–64. They used a time-series forecasting method to project the likely trend in suicide starting in April of 2017, the first full month of the show’s release. Their analysis indicated that compared to the forecast, the rate of suicide deaths in the 10–17 age group exceeded the forecast for the three months following the show’s release and produced an incremental number of suicide deaths for the remainder of the year. Further analysis revealed that the increase was only evident for boys.

The Bridge et al. analysis relied heavily on a forecast of future trends in suicide. I used a straightforward auto-regression analysis that took into account the long-term time trend, autocorrelation, and a discontinuity in the trend following the release of the first season of the show [10]. In order to identify effects of the show, it is necessary to remove trends and autocorrelation. Thus, I first identified secular trends in the 60-month suicide rate series separately for boys and girls that Bridge et al. analyzed from January 2013 to December 2017. After removing those trends from each series, I identified sources of autocorrelation using the partial auto-correlation function. After removing auto-correlation, the resulting series were tested using OLS regression to identify changes in suicide rates for the month of April 2017, the month the show first appeared, and for three months following the release of the show (see the use of similar methods [11]. The Appendix contains all of raw outputs from the analyses conducted using SPSS.

## Results

**Fig 2:**
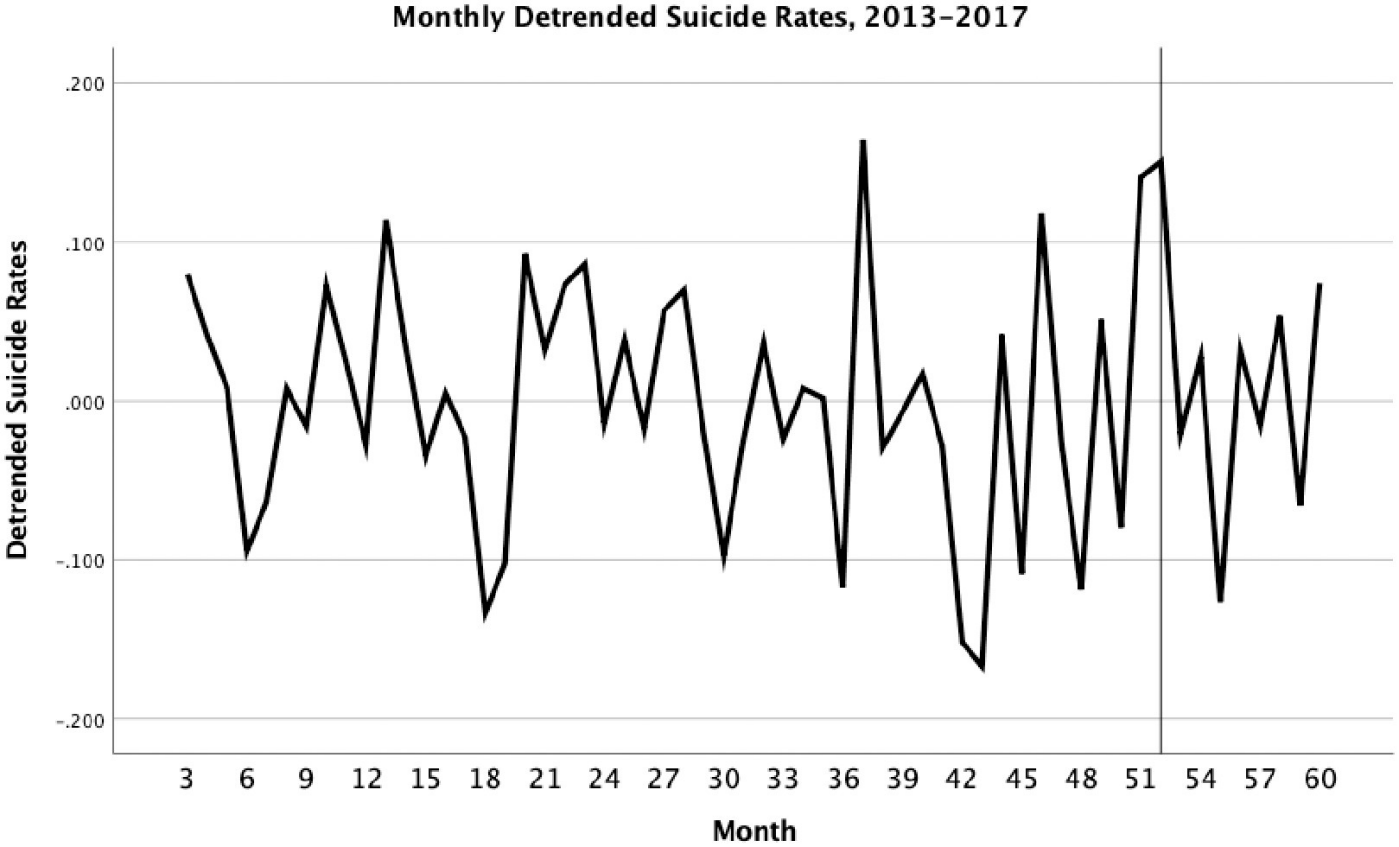
Time series from January 2013 to December 2017 after removal of linear and quadratic trends and autocorrelation in suicide for boys ages 10–17 with month 52 (April of 2017) referenced.

The reanalysis of the Bridge et al. data identified a significant linear and quadratic trend (R^2^ = .366) for suicide in boys just as the trend in Fig 1 would suggest. In addition, there were two lags of autocorrelation in the series. The first-order lag was positive (B = .402, 95% CI = .148, .656) and the second was negative (B = -.305, 95% CI = -.559, -.051). This pattern implies that months one lag apart were positively correlated, and those two lags apart were negatively correlated. As seen in Fig 2, the time series after removal of trends and autocorrelation appears to be stationary with no evidence of change in mean or variance over time. In addition, the increase that Bridge et al. observed in April was replicated, B = .173, 95% CI = .011, .335. However, there was no statistically significant increase for the three months following the release, B = -.040, 95% CI = -.050, .010, contrary to what was reported by Bridge et al. More importantly, there was also a large increase in March before the show was released (see Fig 2). The increase in April was no different statistically from the one in March, B = .006, 95% CI = -.051, .063.

**Fig 3:**
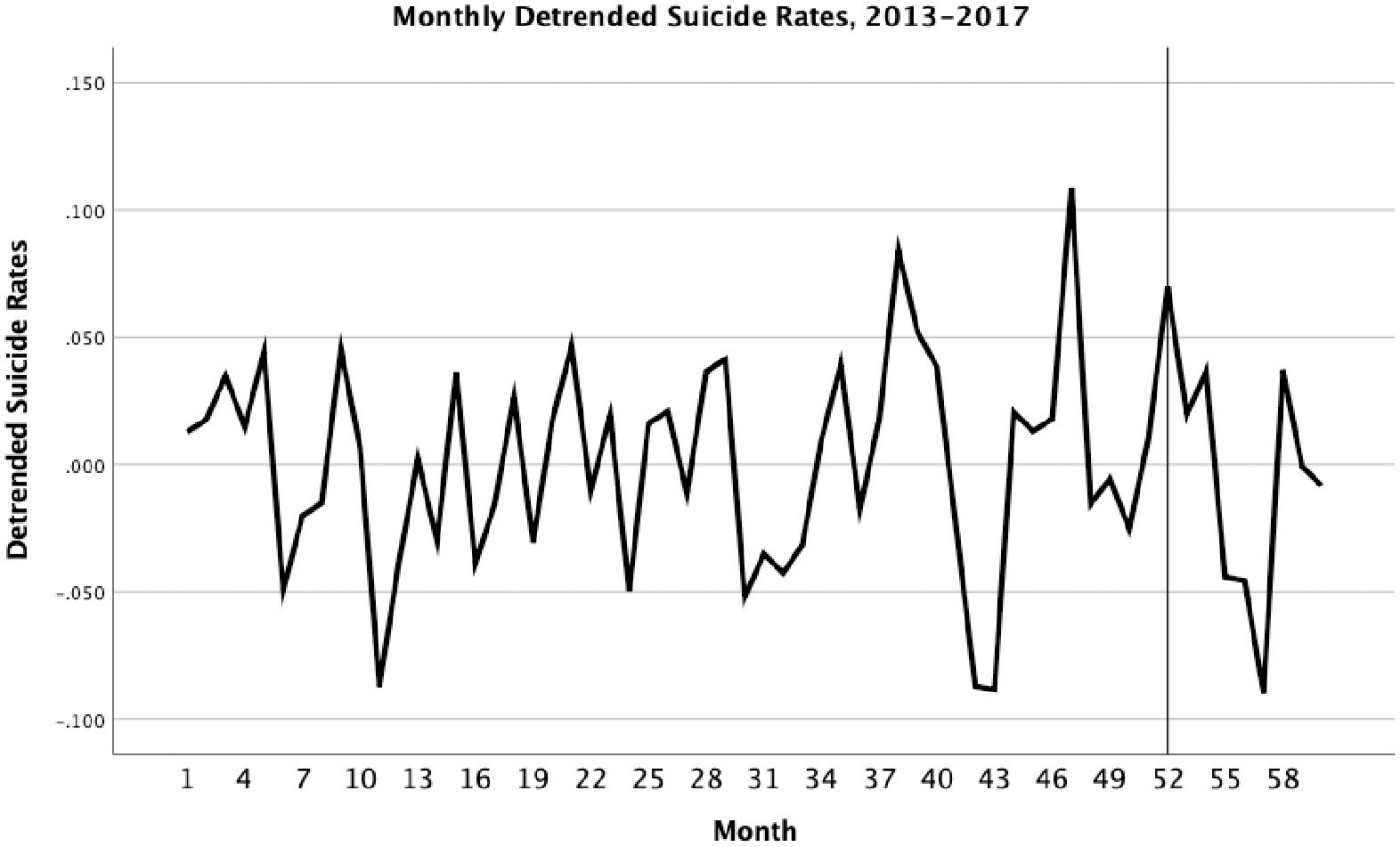
Time series from January 2013 to December 2017 after removal of linear trend in suicide for girls ages 10–17 with month 52 (April of 2017) referenced.

Things were different when we examined the suicide series in girls of the same age. Here we found a significant trend in suicide that was primarily linear in form (R^2^ = .244) as the trends in Fig 1 would suggest. However, there was no significant auto-correlation in the first three lags. We again found a positive but, in this case, non-significant increase in April that was not presaged by an increase in March, B = .073, 95% CI = -.011, .157 (see Fig 3). Girls also displayed many departures from trend during the latter half of this time frame, adding uncertainty to the source of the April 2017 peak.

## Discussion

My analysis of suicide trends examined [1] in boys and girls ages 10 to 17 over a 60-month period raises concerns about attributing contagion effects to the first season of the television series 13RW. This analysis suggests that it is difficult to attribute the rise in male suicide in April 2017 to the show, especially considering that males were not the audience at risk of contagion. Furthermore, the increase in April was not different from the increase that occurred in March before the show was released, again suggesting that other factors were at play in those two months. Finally, Bridge et al. attributed elevations in suicide much past the month of the show’s release, but these changes were more likely attributable to the large increase in suicide observed in boys for the year of 2017, a trend that had started in 2008. Thus, it is equally if not more likely that the rise in those two months was attributable to other sources that were responsible for the large increase in 2017.

Despite the highly variable trend in suicide for girls, the positive but non-statistically significant increase in the month of the show’s release is more convincing of a possible contagion effect than the Bridge et al. finding for boys. This conclusion seems plausible considering that the show was more likely to affect girls adversely even though some girls may have benefited from the show [9]. Our analysis also illustrates the difficulty of separating Werther from Papageno effects in suicide contagion. There may well have been more Werther than Papageno effects of the first season but identifying that effect in aggregate is difficult [12]. Similar concerns are applicable to another study that examined the effects of the first season of 13RW [13].

It is also possible that the effect for girls was more evident in self harm, a behavior that would not necessarily result in more suicide. An analysis of admissions to a hospital in Oklahoma following the release of the show indicated an increase in such behavior [6]. However, here again it would be important to control for trends in this behavior. There is also evidence that admissions to hospital emergency departments for self-injury have increased in recent years [14]. Without controlling for this secular trend and determining gender differences in self harm, it is not possible to confidently attribute changes in self-harm to the show.

One might ask why the Bridge et al. study attributed the April rise in boys to the show. Their analysis used a forecasting procedure to establish a baseline for evaluating changes in suicide in 2017. This forecast was notably insensitive to the secular change in suicide in youth and thus predicted a flat trend for 2017. As a result, their model attributed the increases during 2017 to the show rather than to the secular change. A similar procedure was used [6], which again raises concerns about the conclusions they drew about the show.

Limitations. Because the change in suicide observed for boys occurred one month before the show appeared, it will be important to analyze suicide trends at a more fine-grained level. For example, if weekly suicide rates were available in the US, this would enable one to determine whether the rise that was observed in March continued into the early part of April before the show would have been expected to have its greatest impact. On the other hand, if the March peak occurred early in the month and then subsided before the increase in April, that could suggest a contagion effect after the show appeared. An auto-regression model that takes into account secular trends in weekly suicide may be able to disentangle the effects of the show from other influences for both boys and girls.

In conclusion, I applaud Bridge et al. for analyzing suicide trends following the first season of 13RW. At the same time, I take issue with their analysis which did not take into account the secular trend in suicide and the large increase that occurred in 2017 in young men. Indeed, their analysis essentially identified that departure and attributed it to the show. I also recommend that researchers analyzing trends in time series use a more transparent analytic method that does not depend on unstated assumptions. We used simple auto-regression procedures that make few assumptions and provide robust estimates [10].

For the producers of the Netflix show, their interest in portraying the harmful effects of youth culture, especially on young women, may have had some benefits. But at the same time, it is likely that 13RW had a net effect that was more detrimental to the health and well-being of young vulnerable female viewers. It should certainly be possible to construct a story about these issues that educates without harming its viewers. This is the challenge that 13RW may not have met [9].

## Supporting information

S1 Appendix (DOCX)

S1 Data (SAV)
