# Supplementary material for: Reanalysis of the Bridge et al. study of suicide following release of 13 Reasons Why
Source: PLoS One. 2020 Jan 16;15(1):e0227545. doi: 10.1371/journal.pone.0227545 (PMC6964826; doi:10.1371/journal.pone.0227545)
Supplement: S1 Appendix — (DOCX) [file pone.0227545.s001.docx]

**Appendix**

**Analysis of Male Suicide Rates**

Curve fit with linear and quadratic trend

Analysis showed that quadratic fit was marginally better than linear model, which also corresponds with national trend in male suicides.

Curvefit in SPSS

**Dependent Variable Deaths per Month**

**Linear**

| **Model Summary** | | | |
| --- | --- | --- | --- |
| R | R Square | Adjusted R Square | Std. Error of the Estimate |
| .579 | .335 | .323 | .088 |
| The independent variable is Month. | | | |

| **ANOVA** | | | | | |
| --- | --- | --- | --- | --- | --- |
|  | Sum of Squares | df | Mean Square | F | Sig. |
| Regression | .224 | 1 | .224 | 29.208 | .000 |
| Residual | .444 | 58 | .008 |  |  |
| Total | .668 | 59 |  |  |  |
| The independent variable is Month. | | | | | |

| **Coefficients** | | | | | |
| --- | --- | --- | --- | --- | --- |
|  | Unstandardized Coefficients | | Standardized Coefficients | t | Sig. |
|  | B | Std. Error | Beta |  |  |
| Month | .004 | .001 | .579 | 5.404 | .000 |
| (Constant) | .391 | .023 |  | 17.081 | .000 |

**Quadratic**

| **Model Summary** | | | |
| --- | --- | --- | --- |
| R | R Square | Adjusted R Square | Std. Error of the Estimate |
| .605 | .366 | .344 | .086 |
| The independent variable is Month. | | | |

| **ANOVA** | | | | | |
| --- | --- | --- | --- | --- | --- |
|  | Sum of Squares | df | Mean Square | F | Sig. |
| Regression | .244 | 2 | .122 | 16.463 | .000 |
| Residual | .423 | 57 | .007 |  |  |
| Total | .668 | 59 |  |  |  |
| The independent variable is Month. | | | | | |

| **Coefficients** | | | | | |
| --- | --- | --- | --- | --- | --- |
|  | Unstandardized Coefficients | | Standardized Coefficients | t | Sig. |
|  | B | Std. Error | Beta |  |  |
| Month | -.001 | .003 | -.117 | -.274 | .785 |
| Month ** 2 | 6.951E-5 | .000 | .718 | 1.675 | .099 |
| (Constant) | .435 | .035 |  | 12.591 | .000 |


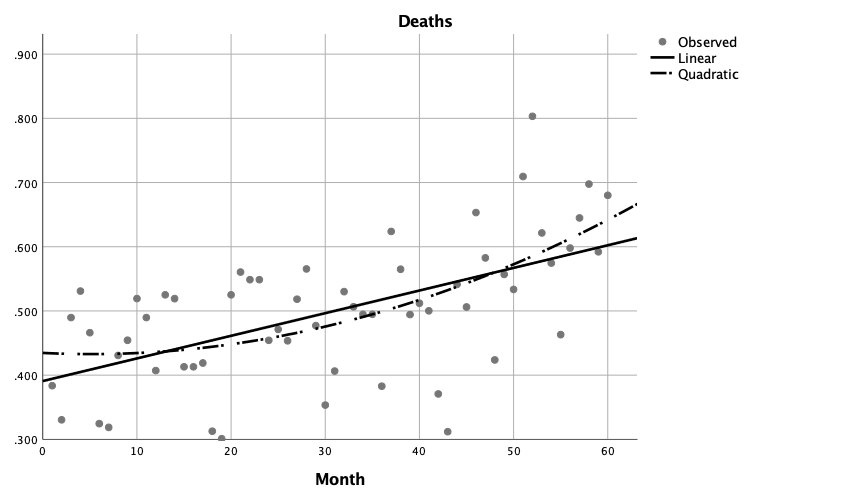


Test for autocorrelation in residuals after removal of trends revealed two significant lags

| **Partial Autocorrelations** | | |
| --- | --- | --- |
| Series: Error for Deaths with Month from CURVEFIT, MOD_5 QUADRATIC | | |
| Lag | Partial Autocorrelation | Std. Error |
| 1 | .314 | .129 |
| 2 | -.304 | .129 |
| 3 | -.193 | .129 |
| 4 | -.177 | .129 |
| 5 | .126 | .129 |
| 6 | -.037 | .129 |
| 7 | -.059 | .129 |
| 8 | -.226 | .129 |
| 9 | -.013 | .129 |
| 10 | -.163 | .129 |
| 11 | .027 | .129 |
| 12 | .156 | .129 |
| 13 | -.101 | .129 |
| 14 | .105 | .129 |
| 15 | -.109 | .129 |
| 16 | .035 | .129 |


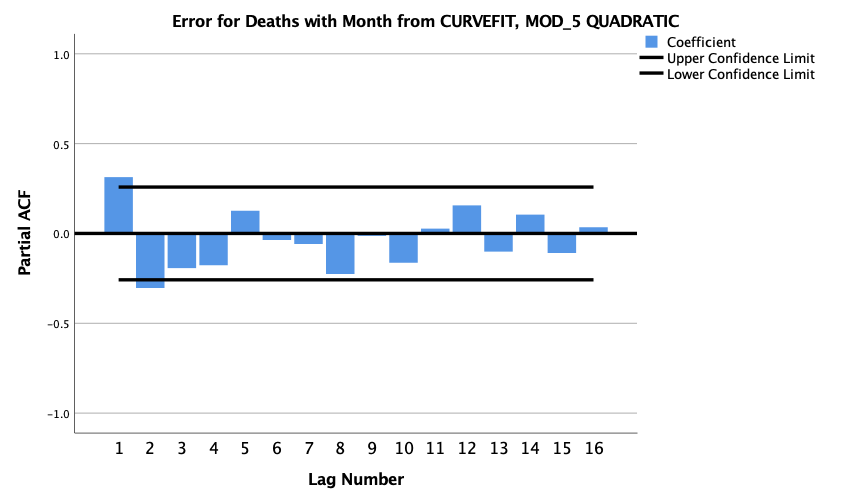


After removing first two lags of autocorrelation, examined the distribution of residuals prior to conducting tests of changes in rates starting in month 52 (April of 2017). No evidence of departure from normality.

**Frequencies**

| **Statistics** | | |
| --- | --- | --- |
| Residual after autocorrelation removal | | |
| N | Valid | 58 |
|  | Missing | 4 |
| Skewness | | -.122 |
| Std. Error of Skewness | | .314 |
| Kurtosis | | -.308 |
| Std. Error of Kurtosis | | .618 |


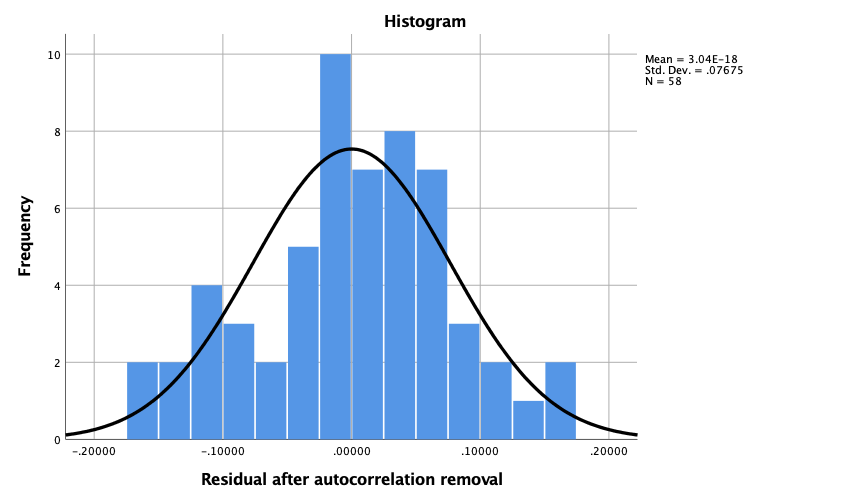


Test of month 52 change in suicide rates

| **Model Summary** | | | | |
| --- | --- | --- | --- | --- |
| Model | R | R Square | Adjusted R Square | Std. Error of the Estimate |
| 1 | .049^a^ | .002 | -.034 | .07803733 |
| a. Predictors: (Constant), Month 52, Month | | | | |

| **ANOVA^a^** | | | | | | |
| --- | --- | --- | --- | --- | --- | --- |
| Model | | Sum of Squares | df | Mean Square | F | Sig. |
| 1 | Regression | .001 | 2 | .000 | .066 | .936^b^ |
|  | Residual | .335 | 55 | .006 |  |  |
|  | Total | .336 | 57 |  |  |  |
| a. Dependent Variable: Residual after autocorrelation removal | | | | | | |
| b. Predictors: (Constant), Month 52, Month | | | | | | |

| **Coefficients^a^** | | | | | | |
| --- | --- | --- | --- | --- | --- | --- |
| Model | | Unstandardized Coefficients | | Standardized Coefficients | t | Sig. |
|  |  | B | Std. Error | Beta |  |  |
| 1 | (Constant) | .007 | .022 |  | .311 | .757 |
|  | Month | .000 | .001 | -.047 | -.353 | .726 |
|  | Month 52 | .005 | .055 | .013 | .094 | .926 |
| a. Dependent Variable: Residual after autocorrelation removal | | | | | | |

Test of effect for months of April, May, June (Spring)

Test of months April, May, June (Spring)

| **Model Summary** | | | | |
| --- | --- | --- | --- | --- |
| Model | R | R Square | Adjusted R Square | Std. Error of the Estimate |
| 1 | .148^a^ | .022 | -.032 | .07798497 |
| a. Predictors: (Constant), spring, Month, Month 52 | | | | |

| **ANOVA^a^** | | | | | | |
| --- | --- | --- | --- | --- | --- | --- |
| Model | | Sum of Squares | df | Mean Square | F | Sig. |
| 1 | Regression | .007 | 3 | .002 | .402 | .752^b^ |
|  | Residual | .328 | 54 | .006 |  |  |
|  | Total | .336 | 57 |  |  |  |
| a. Dependent Variable: Residual after autocorrelation removal | | | | | | |
| b. Predictors: (Constant), spring, Month, Month 52 | | | | | | |

| **Coefficients^a^** | | | | | | |
| --- | --- | --- | --- | --- | --- | --- |
| Model | | Unstandardized Coefficients | | Standardized Coefficients | t | Sig. |
|  |  | B | Std. Error | Beta |  |  |
| 1 | (Constant) | .015 | .023 |  | .649 | .519 |
|  | Month | .000 | .001 | -.060 | -.446 | .657 |
|  | Month 52 | .018 | .056 | .043 | .313 | .756 |
|  | spring | -.025 | .024 | -.143 | -1.036 | .305 |
| a. Dependent Variable: Residual after autocorrelation removal | | | | | | |

Test of contrast between month 51 and month 52

| **Model Summary** | | | | |
| --- | --- | --- | --- | --- |
| Model | R | R Square | Adjusted R Square | Std. Error of the Estimate |
| 1 | .049^a^ | .002 | -.034 | .07803733 |
| a. Predictors: (Constant), contrast, Month | | | | |

| **ANOVA^a^** | | | | | | |
| --- | --- | --- | --- | --- | --- | --- |
| Model | | Sum of Squares | df | Mean Square | F | Sig. |
| 1 | Regression | .001 | 2 | .000 | .066 | .936^b^ |
|  | Residual | .335 | 55 | .006 |  |  |
|  | Total | .336 | 57 |  |  |  |
| a. Dependent Variable: Residual after autocorrelation removal | | | | | | |
| b. Predictors: (Constant), contrast, Month | | | | | | |

| **Coefficients^a^** | | | | | | |
| --- | --- | --- | --- | --- | --- | --- |
| Model | | Unstandardized Coefficients | | Standardized Coefficients | t | Sig. |
|  |  | B | Std. Error | Beta |  |  |
| 1 | (Constant) | .007 | .022 |  | .311 | .757 |
|  | Month | .000 | .001 | -.047 | -.353 | .726 |
|  | Contrast | .005 | .055 | .013 | .094 | .926 |
| a. Dependent Variable: Residual after autocorrelation removal | | | | | | |

**Analysis of Female Suicide Rates**

Analysis of trend revealed only a linear component, consistent with national trends for females

**Linear**

| **Model Summary** | | | |
| --- | --- | --- | --- |
| R | R Square | Adjusted R Square | Std. Error of the Estimate |
| .494 | .244 | .231 | .042 |
| The independent variable is Month. | | | |

| **ANOVA** | | | | | |
| --- | --- | --- | --- | --- | --- |
|  | Sum of Squares | df | Mean Square | F | Sig. |
| Regression | .033 | 1 | .033 | 18.744 | .000 |
| Residual | .103 | 58 | .002 |  |  |
| Total | .137 | 59 |  |  |  |
| The independent variable is Month. | | | | | |

| **Coefficients** | | | | | |
| --- | --- | --- | --- | --- | --- |
|  | Unstandardized Coefficients | | Standardized Coefficients | t | Sig. |
|  | B | Std. Error | Beta |  |  |
| Month | .001 | .000 | .494 | 4.329 | .000 |
| (Constant) | .183 | .011 |  | 16.624 | .000 |

**Quadratic**

| **Model Summary** | | | |
| --- | --- | --- | --- |
| R | R Square | Adjusted R Square | Std. Error of the Estimate |
| .494 | .244 | .218 | .043 |
| The independent variable is Month. | | | |

| **ANOVA** | | | | | |
| --- | --- | --- | --- | --- | --- |
|  | Sum of Squares | df | Mean Square | F | Sig. |
| Regression | .033 | 2 | .017 | 9.217 | .000 |
| Residual | .103 | 57 | .002 |  |  |
| Total | .137 | 59 |  |  |  |
| The independent variable is Month. | | | | | |

| **Coefficients** | | | | | |
| --- | --- | --- | --- | --- | --- |
|  | Unstandardized Coefficients | | Standardized Coefficients | t | Sig. |
|  | B | Std. Error | Beta |  |  |
| Month | .001 | .001 | .540 | 1.154 | .253 |
| Month ** 2 | -2.080E-6 | .000 | -.047 | -.101 | .920 |
| (Constant) | .182 | .017 |  | 10.681 | .000 |


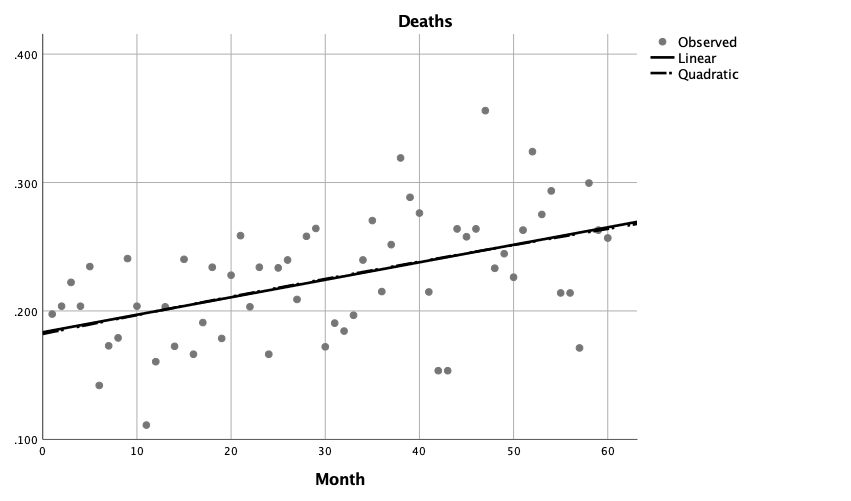


Examination of distribution of residuals revealed no departure from normality

| **Statistics** | | |
| --- | --- | --- |
| Unstandardized Residual | | |
| N | Valid | 60 |
|  | Missing | 4 |
| Skewness | | -.134 |
| Std. Error of Skewness | | .309 |
| Kurtosis | | .110 |
| Std. Error of Kurtosis | | .608 |


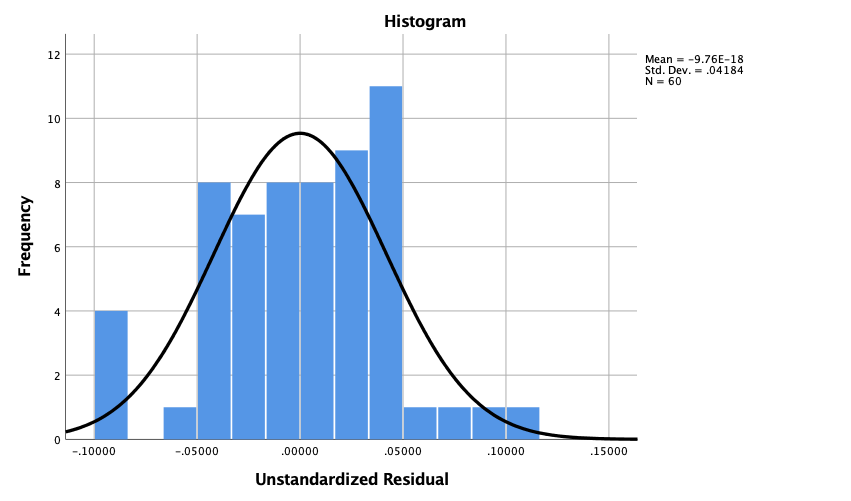


Test of autocorrelation revealed no significant lags except for 4^th^. Did not pursue this lag.

| **Partial Autocorrelations** | | |
| --- | --- | --- |
| Series: Unstandardized Residual | | |
| Lag | Partial Autocorrelation | Std. Error |
| 1 | .199 | .129 |
| 2 | -.094 | .129 |
| 3 | -.184 | .129 |
| 4 | -.302 | .129 |
| 5 | .010 | .129 |
| 6 | .009 | .129 |
| 7 | -.110 | .129 |
| 8 | -.108 | .129 |
| 9 | -.120 | .129 |
| 10 | -.129 | .129 |
| 11 | -.034 | .129 |
| 12 | .005 | .129 |
| 13 | .002 | .129 |
| 14 | -.046 | .129 |
| 15 | -.104 | .129 |
| 16 | .043 | .129 |


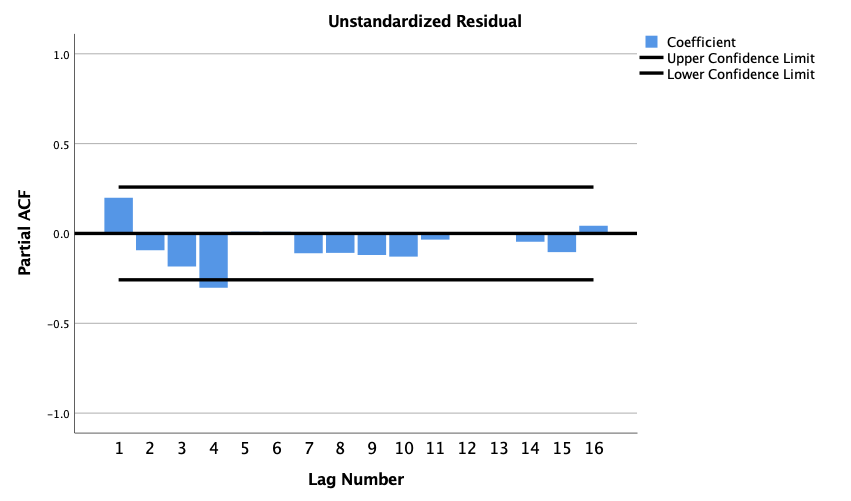


Test for month 52

| **Model Summary** | | | | |
| --- | --- | --- | --- | --- |
| Model | R | R Square | Adjusted R Square | Std. Error of the Estimate |
| 1 | .222^a^ | .049 | .016 | .04150516 |
| a. Predictors: (Constant), high1, Month | | | | |

| **ANOVA^a^** | | | | | | |
| --- | --- | --- | --- | --- | --- | --- |
| Model | | Sum of Squares | df | Mean Square | F | Sig. |
| 1 | Regression | .005 | 2 | .003 | 1.478 | .237^b^ |
|  | Residual | .098 | 57 | .002 |  |  |
|  | Total | .103 | 59 |  |  |  |
| a. Dependent Variable: Unstandardized Residual | | | | | | |
| b. Predictors: (Constant), high1, Month | | | | | | |

| **Coefficients^a^** | | | | | | |
| --- | --- | --- | --- | --- | --- | --- |
| Model | | Unstandardized Coefficients | | Standardized Coefficients | t | Sig. |
|  |  | B | Std. Error | Beta |  |  |
| 1 | (Constant) | .001 | .011 |  | .132 | .895 |
|  | Month | -8.711E-5 | .000 | -.036 | -.278 | .782 |
|  | high1 | .073 | .042 | .225 | 1.719 | .091 |
| a. Dependent Variable: Unstandardized Residual | | | | | | |

Test for months of April, May, June (Spring)

| **Model Summary** | | | | |
| --- | --- | --- | --- | --- |
| Model | R | R Square | Adjusted R Square | Std. Error of the Estimate |
| 1 | .222^a^ | .049 | -.002 | .04187396 |
| a. Predictors: (Constant), spring, Month, high1 | | | | |

| **ANOVA^a^** | | | | | | |
| --- | --- | --- | --- | --- | --- | --- |
| Model | | Sum of Squares | df | Mean Square | F | Sig. |
| 1 | Regression | .005 | 3 | .002 | .968 | .414^b^ |
|  | Residual | .098 | 56 | .002 |  |  |
|  | Total | .103 | 59 |  |  |  |
| a. Dependent Variable: Unstandardized Residual | | | | | | |
| b. Predictors: (Constant), spring, Month, high1 | | | | | | |

| **Coefficients^a^** | | | | | | |
| --- | --- | --- | --- | --- | --- | --- |
| Model | | Unstandardized Coefficients | | Standardized Coefficients | t | Sig. |
|  |  | B | Std. Error | Beta |  |  |
| 1 | (Constant) | .001 | .012 |  | .117 | .907 |
|  | Month | -8.656E-5 | .000 | -.036 | -.273 | .786 |
|  | high1 | .073 | .044 | .224 | 1.651 | .104 |
|  | spring | .000 | .013 | .003 | .020 | .984 |
| a. Dependent Variable: Unstandardized Residual | | | | | | |
